# Idiosyncratic Drug Induced Liver Injury, Cytochrome P450, Metabolic Risk Factors and Lipophilicity: Highlights and Controversies

**DOI:** 10.3390/ijms22073441

**Published:** 2021-03-26

**Authors:** Rolf Teschke, Gaby Danan

**Affiliations:** 1 Department of Internal Medicine II, Division of Gastroenterology and Hepatology, Klinikum Hanau, D-63450 Hanau, Academic Teaching Hospital of the Medical Faculty, Goethe University Frankfurt/Main, 60323 Frankfurt/Main, Germany; 2 Pharmacovigilance Consultancy, F-75020 Paris, France; gaby.danan@gmail.com

**Keywords:** cytochrome P450, CYP isoforms, DILI, daily drug dose, iDILI, idiosyncratic drug induced liver injury, drug metabolism, lipophilicity, metabolic risk factors, reactive oxygen species (ROS), Roussel Uclaf Causality Assessment Method, RUCAM

## Abstract

Progress in understanding the mechanisms of the idiosyncratic drug induced liver injury (iDILI) was highlighted in a scientometric investigation on the knowledge mapping of iDILI throughout the world, but uncertainty remained on metabolic risk factors of iDILI, the focus of the present review article. For the first time, a quantitative analysis of 3312 cases of iDILI assessed for causality with RUCAM (Roussel Uclaf Causality Assessment Method) showed that most drugs (61.1%) were metabolized by cytochrome P450 (CYP) isoforms: 49.6% by CYP 3A4/5, 24.6% by CYP 2C9, 13.2% by CYP 2E1, 7.3% by CYP 2C19, 3.5% by CYP 1A2 and 1.8% by CYP 2D6. Other studies showed high OR (odds ratio) for drugs metabolized by unspecified CYPs but the iDILI cases were not assessed for causality with RUCAM, a major shortcoming. In addition to critical comments on methodological flaws, several risk factors of iDILI were identified such as high but yet recommended daily drug doses, actual daily drug doses taken by the patients, hepatic drug metabolism and drug lipophilicity. These risk factors are subject to controversies by many experts seen critically also by others who outlined that none of these medication characteristics is able to predict iDILI with high confidence, leading to the statement of an outstanding caveat. It was also argued that all previous studies lacked comprehensive data because the number of examined drugs was relatively small as compared to the number of approved new molecular entities or currently used oral prescription drugs. In conclusion, trends are evident that some metabolic parameters are likely risk factors of iDILI but strong evidence can only be achieved when methodological issues will be successfully met.

## 1. Introduction

Idiosyncratic drug induced liver injury (iDILI) was the focus of three recent publications dealing with specific issues [1,2,3]. The first one was the analysis of 81,856 published cases of iDILI assessed for causality by the Roussel Uclaf Causality Assessment Method (RUCAM) [1]. The second one reported on the scientometric study on the knowledge mapping of iDILI throughout the world with details on the most quoted publications and scientists most engaged in research [2]. The third one was focused on 3312 published iDILI cases assessed for causality with RUCAM in order to establish a list of drugs most implicated in iDILI [3]. Among the top 10 drugs were amoxicillin-clavulanate, flucloxacillin, atorvastatin, disulfiram, diclofenac, simvastatin, carbamazepine, ibuprofen, erythromycin and anabolic steroids as body building agents. This ranking would likely reflect the extent of the drug use and probably not the strength of their hepatotoxicity. The latter can only be determined in the same geographic area where the number of iDILI cases and the drug exposure can be measured.

The identification of risk factors and description of mechanistic steps leading to iDILI were outlined in other reports [4,5]. The idiosyncratic nature of the liver injury means that the liver injury is specific to a patient and therefore is unpredictable, rare and not easily reproducible in animal models [4]. These characteristics make it difficult to establish risk factors based on pathogenetic principles, that would add to problems extrapolating animal results to human disease [4,5]. More challenging are studies in humans because the liver is known as a secret keeping organ, hardly accessible [5] and, more importantly, patients with iDILI evaluated by a robust causality assessment method (CAM) such as RUCAM are rarely available as a homogenous study cohort in one place. Because the liver is the central organ for drug metabolism another approach to clarify iDILI features would be to look more closely on the products of metabolic events as potential risk factors, which must be eliminated from the body to prevent deleterious effects due to accumulation. Of great and well-known interest are hepatic microsomal cytochrome P450 (CYP) isoforms, the rate of drug metabolism, the actual used daily drug dose (UDDD) versus the recommended daily drug dose (RDDD), cumulative drug dose (CDD) and drug lipophilicity (DL) in patients with iDILI, but uncertainty remains if the cases had not been assessed for causality by RUCAM [6,7,8] or any other robust CAM.

In this review, the relationship between iDILI and hepatic pathways of drug metabolism is analysed with focus on potential risk factors, a topic controversially discussed in the literature due to inconsistent data. The difficulties could mostly be explained by differences in methodology including variability of case sources and data quality. In addition, a broad range of drugs are potentially hepatotoxic and may lead to variable clinical features.

## 2. Literature Search and Source

The PubMed database was searched for articles by using the following key terms: idiosyncratic drug induced liver injury (DILI); drugs; cytochrome P450, CYP. These terms were used alone or in combination. Limited to the English language, publications from each search terms were analyzed for suitability of this review article. Publications were complemented from the large private archives of the authors. The final compilation consisted of original papers, consensus reports and review articles with the most relevant publications included in the reference list of this review.

## 3. Pathways of Hepatic Drug Metabolism

The liver is exposed to high concentrations of drugs and metabolites after oral administration [9], based on the view that the portal blood brings drugs and xenobiotics absorbed by the gut directly to the liver in high concentrations [10]. The drug concentration in the hepatocytes is influenced by the relative speed of drug uptake, metabolism and excretion [11,12,13]. Among the mechanisms usually described in iDILI are: first, the passive drug diffusion from the blood or active drug influx mechanisms via transporters such as NTCP (Na+-taurocholate cotransporting polypeptide), OCT (organic cation transporter) and OATP (organic anion transporting polypeptide), these processes are localized in the sinusoidal plasma membrane of the hepatocyte [11]. Second, and more importantly, drug biotransformation in the liver cell by metabolizing enzymes such as CYP isoforms [11,12] or nonCYP pathways like flavin-containing monooxygenase (FMO), monoamine oxidase (MAO), alcohol dehydrogenase (ADH), acetaldehyde dehydrogenase (ALDH) and aldehyde oxidase (AO) [11,13], grouped as phase I reactions involving oxidation, reduction, or hydrolysis [11,13] and/or via conjugating enzymes grouped as phase II reactions [11], including UDP-glycosyltransferase (UGT), glutathione S-transferase (GST), sulfotransferase (SULT) and N-acetyltransferase (NAT) [11,13]. Third, the elimination of the parent drug or its metabolites occurs preferentially via the bile canalicular pole of the plasma membrane of the hepatocyte by drug efflux mechanisms through transporters like BSEP (bile salt export pump), BCRP (breast cancer resistance protein), MDR (multidrug resistance protein) and MRP (multidrug resistance-associated protein) [11]. Several hundred drugs can induce iDILI [14], which makes it difficult to assign for each drug reaction an individual mechanism of liver injury although several attempts were made in this area [4,5,13,15,16,17,18,19,20]. Despite the abundance of metabolic pathways involved in drug biotransformation in the liver, most important pathways involve CYPs isoforms (Table 1) [21].

## 4. Cytochrome P450 

**Figure 1:**
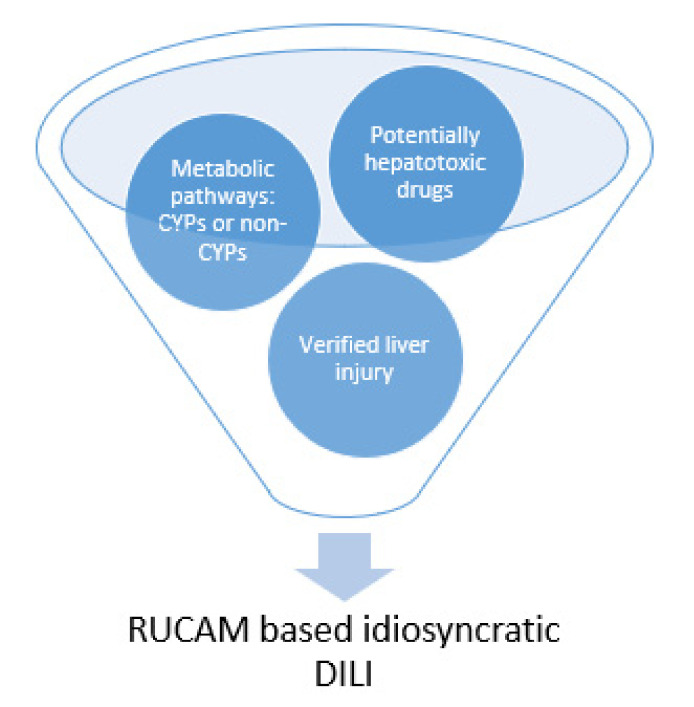
Metabolic pathways involved in liver injury caused by drugs. Potentially hepatotoxic drugs are commonly eliminated in the liver via metabolic pathways involving cytochrome P450 (CYP) isoforms of nonCYP dependent enzymes. Liver injury must be verified, before idiosyncratic drug induced liver injury (DILI) can be established as valid diagnosis requiring causality assessment with the updated RUCAM (Roussel Uclaf Causality Assessment Method) [8].

Among potentially hepatotoxic drugs, most are metabolized by CYPs and to a lesser extent through pathways involving nonCYP enzymes. Independent from the metabolic pathway, the liver injury has to be verified using specific threshold criteria of ALT and/or ALP associated with the causality assessment using the RUCAM (Figure 1) in its updated version [8].

### CYP Dependent Drugs and iDILI

Few reports discussed the possible role of CYP dependent drug metabolism triggering the initiation and perpetuation of iDILI (Table 1) [9,18,21]. For instance, it was assumed that drugs implicated in iDILI were mainly metabolized by pathways dependent on the action of CYPs [18]. However, this assumption was not evidence based on high quality data of iDILI cases assessed for causality with a robust CAM such as RUCAM although this diagnostic algorithm was referenced, validated and widely used [18]. Similarly, the US FDA (Food and Drug Administration) was referenced as officially stating in 2009, without providing any evidence that most hepatotoxic drugs were oxidatively metabolized by the CYP systems [9]. The results of another study on 254 drugs suggested that being a substrate of CYP enzymes is one of two important predictors of iDILI, based on the adjusted OR (odds ratio) of 5.04 (95% confidence interval (CI) 2.34–10.9, p <0.0001) [9]. In this study, the data on the metabolism of the administered drugs involving CYP enzymes derived from the Liver Toxicity Knowledge Base Benchmark Dataset, where the case data quality cannot be assessed namely on the use of RUCAM or any other CAM [9]. Data were also presented of 11 clinical iDILI cases retrieved from the US LiverTox database with causality assessment confirmed by health care professionals [9]. As a reminder, many iDILI cases of this database were not assessed by validated CAMs and became a matter of serious debate [3,27,28,29]. Other iDILI cases derived from Liver Toxicity Biomarker Study lack details on causality assessment [9]. Considering the poor documentation on data quality [9], the results should be qualified as preliminary at best. On a quantitative basis, it was for the first time in 2020 (Table 1) that CYP involvement in the metabolism of drugs implicated in iDILI was appropriately determined (Table 2) [3,5,21].

To quantify the role of CYPs in iDILI, 36 top drugs implicated in cases of iDILI assessed for causality using RUCAM were selected for analysis [3,5,21]. Among the 36 drugs, 22 drugs (61.1%) were metabolized through CYP pathways whereas 14 drugs (38.9%) were metabolized via other pathways (Table 2) [21]. As a result, drugs might cause iDILI independently from the metabolic pathways, defining two different cohorts, termed as CYP dependent iDILI and CYP independent iDILI [21]. Currently, it is unknown as to whether these two iDILI cohorts show differences in terms of mechanistic steps, laboratory data, clinical features and prognosis.

Clarification of the potential role of CYP isoforms in iDILI was presented in a recent study (Table 2) [21]. The CYP dependent iDILI cohort consisted of 619 cases caused by 22 drugs metabolized by 6 CYP isoforms (Table 2). Almost half (49.1%) of these cases were caused by drugs metabolized by CYP 3A4/5 and almost a quarter (24.6%) by CYP 2C8/9, the remaining quarter by the CYP isoforms CYP 2E1, CYP 2C19, CYP 1A2 and CYP 2D6 (Table 2). The comparison of these figures with the distribution of CYP isoforms in a healthy population [12] reveals that in the iDILI group there were more drugs metabolized by CYP 2C9 and CYP 2E1 and less drugs metabolized via CYP 2D6 (Table 3).

Previous studies suggested that drugs metabolized by CYP 1A2, CYP 2C8/2C9 and CYP 3A5 have a higher likelihood of causing iDILI, based on 254 drugs and iDILI cases not assessed for causality with RUCAM and collected from the US Liver Toxicity Knowledge Base Benchmark Dataset, lacking quality details on the cases [9]. These results are at variance with the data obtained from 619 drugs implicated in RUCAM based iDILI cases [21]. The differences could partially be explained by the low drug case numbers [9] versus the high drug numbers [21] and by iDILI cases not assessed for causality by RUCAM [9] versus RUCAM based iDILI cases [21]. In addition, another study showed that drugs metabolized by CYP 2C9 and CYP 2C19 were at a higher risk of causing iDILI as compared with drugs metabolized through CYP 3A and CYP 2D6 pathways [22]. It is interesting to note that each of the 3 studies [9,21,22] attributed the risk level of various CYP isoforms for iDILI differently (Table 4).

Overall, without additional data, it would be premature to classify drugs as low or high risk for iDILI according to CYP isoforms, only because of contradictory data (Table 4). Further studies on CYP isoforms are warranted and should be based on an iDILI cohort assessed for causality by the updated RUCAM [8]. This could allow for a convincing relation between CYP isoforms involved in drug metabolism and the risk of developing iDILI although several CYP isoforms can be involved in the metabolism of one drug making hard conclusions on one CYP as the trigger of iDILI. Available study cohorts lack homogeneity and robust causality assessment of iDILI cases (Table 4), a problem that could be solved in new studies. However, part of the CYP isoforms variability might be caused by factors related to genetic disposition of patients exhibiting differences in CYP isoforms features like Hispanics who have about twice the activity of CYP 2C compared with Caucasians [9]. There is also a gender difference, with a twofold higher activity of CYP 3A4 in the liver of women compared with that of men. None of these variables have been considered in the reports [9,21,22], preventing any definitive conclusions.

Poorly understood are the mechanistic steps whereby CYP isoforms trigger and perpetuate iDILI [5]. Suggestions have been made that reactive metabolites like in acetaminophen intrinsic toxicity and/or reactive oxygen species (ROS) generated during the catalytic CYP cycle are involved in triggering iDILI by these drugs [21]. In this context, ROS could modify directly cytosol or membrane cell proteins or indirectly hepatic RNA, which after activation could code for proteins functioning as antigens and activating the adaptive immune system. Characterized by immunological features including CYP antibody generation, liver injury by halothane is an example of iDILI caused by a drug metabolized by CYP 2E1 [21,30]. Using iDILI cases caused by other drugs and assessed by the updated RUCAM [8], further clinical studies are needed to verify or dismiss this hypothesis.

## 5. Recommended Daily Drug Dose (RDDD)

Daily drug doses need differentiation of recommended dose ranges provided by the clinical studies from doses actually used by the patient with iDILI. Neglecting this gap could lead to false conclusions if, for instance, a high daily drug dose is claimed being at a high risk for iDILI while the high daily dose was merely based on the recommended dose with a broad range rather than on the actual drug dose used by the patient (Table 1). From studies on herb induced liver injury (HILI) by kava, it is known that patients do not necessarily adhere on the recommended daily dose, the maximum recommended treatment duration, or both [31,32]. Similar shortcomings may be found in iDILI patients under real life conditions, not documented in the iDILI databases.

In general, the use of iDILI databases presenting the recommended daily drug doses only with a broad range should be discouraged (Table 1). These shortcomings are frequently combined with a lack of RUCAM use to assess causality of the iDILI cases, conditions that further complicate a correct conclusion.

A relationship was assumed between recommended, not necessarily actually used, daily drug doses of oral medications and iDILI [23]. The US study cohort retrieved the cases and the used drugs including their recommended daily doses from two publicly available pharmaceutical databases. The drugs were categorized in dosage groups of 10 mg or less, 11 to 49 mg and 50 mg or greater based on daily recommended doses, which originally may show a broad range not allowing for inclusion in a precise dosage group. With atorvastatin as an example of a problematic dosage group attribution, the recommended daily dose was described as ranging between 10 and 80 mg. Among US prescription medicines, a statistically significant relationship was observed between recommended daily drug dose and reported frequency of hepatic adverse events like liver failure, liver transplantation and death caused by iDILI. Some data were also used from another report after recalculation of the published results, but iDILI cases were not assessed for causality with RUCAM, a major shortcoming that made questionable the identification of iDILI cases [33]. As a result, the proposed daily drug dose dependency of iDILI was not based on the actually used daily dosage but on drugs with a broad recommended dose range. Therefore, problems of doses and iDILI case quality reduce the validity of the conclusions, classifying the data as preliminary at best.

## 6. Used Daily Drug Dose (UDDD)

Earlier reports arbitrarily classified a drug with a daily dose of 10 mg or less as having no risk of iDILI, opposing to daily drug doses above 10 mg carrying a risk of iDILI (Table 1). There was, however, some confusion since these claims were not based on actual data derived from an own research but resulted from incorrect interpretation of previous reports published in 1999 [20] and 2007 [19]. Indeed, published statements referred to two idiosyncratic drug reactions (iDRs) but not specifically to iDILI cases [19,20]. Only in 2019, iDILI was mentioned in this context but again not based on any iDILI case analysis [4].

Clarification was attempted in a Swedish cohort [23] including 598 iDILI cases, 9% belonged to the ≤10 mg/day group, 14.3% to the 11–49 mg/day group and 77% of cases were caused by medications given at doses ≥50 mg/day [23]. Cases were selected in the Swedish Drug Reaction Advisory Committee (SADRAC) database and included in the cohort when causality was assessed with RUCAM as “possible”. However, the percentage of cases with “possible” causality grading in the entire iDILI cohort was not mentioned [23]. In earlier studies using cases of SADRAC, up to 48% of the iDILI cases caused by statins had a possible causality grading [33], a major shortcoming that became a matter of debate [34,35,36,37,38,39] around the myth of severe liver injury caused by statins [40]. Therefore, these data should be taken with caution, because the number of cases with causality gradings of “highly probable” or “probable” the only ones that should have been taken into consideration remains unclear. The number of cases of clinically significant liver abnormalities with alanine aminotransferase (ALT) values >5 x ULN (upper limit of normal) and whether the cases with ALT values of 2–5 x ULN were excluded from the study cohort is also unclear.

## 7. Cumulative Drug Dose (CDD)

Cumulative doses of drugs implicated in iDILI as a possible risk factor was not considered in any of the published reports (Table 1). Calculations from raw data presented in a single publication [23] showed a broad range of cumulative doses, not suitable to determine a threshold value as a risk factor (Table 5).

Problems were the low number of cases retrieved from the Swedish Hepatic ADR Dataset (Table 6), the questionable inclusion criterion of iDILI cases with possible causality gradings and the low ALT threshold of >2 x ULN (Table 1) [24] that includes non-clinically significant liver injury and therefore reduces the specificity of iDILI cases. Calculating cumulative doses requires precise data on duration of drug use and actually used daily drug dose, not the recommended doses as often presented in iDILI databases.

## 8. Hepatic Drug Metabolism (HDM)

Oral medications with more than 50% of hepatic metabolism were considered at high risk of severe injury (Table 1). However, this statement is subject to discussion due to substantial methodological issues including lack of using RUCAM to identify iDILI cases, thereby limiting the value of the conclusions [22]. Similar disputable conclusions were presented in 2 other reports with problems of iDILI cases not assessed with RUCAM for causality [25,26], considering the extent of hepatic drug metabolism not as a strong predictor for iDILI risk.

## 9. Drug Lipophilicity (DL)

Despite attempts to identify high drug lipophilicity as risk factor of iDILI (Table 1) [24,26], the quality of the presented data was not convincing and remained a matter of debate even among FDA members (Table 1) [9,24,25,26]. In detail, for the odd ratio of drug lipophilicity a statistically significant difference was not found (Table 1) [24]. The number of used cases was considered as low, the quality of the databases from which the cases were retrieved remained unknown, own clinical cases were not included in the studies [24,26] and causality of the used iDILI cases was not assessed with a transparent robust objective diagnostic algorithm like RUCAM (Table 1) [9,25]. Most disturbing was the use of cases retrieved from the US LiverTox database (Table 1) [26], which is known for keeping iDILI cases without diagnostic verification [27,28]. Of note, weaknesses were seen not only for lipophilicity [25] but also for the other parameters as listed (Table 1). It was argued as a caveat that the general belief remains that none of these drug characteristics are able to predict iDILI with a high confidence [25]. Despite this, caveat [25], hope of potential progress and steps forward was announced [26]. Indeed, to establish causality, the data needs to be evaluated by accepted methods of causality assessment such as RUCAM [26].

## 10. Use of RUCAM

In the context of risk factors and mechanistic steps of iDILI it was recently emphasized that the first challenge is the diagnosis of iDILI because it can mimic any other types of liver injury [41]. If cases are misdiagnosed, it can lead to false conclusions about drugs that can cause iDILI and what are the characteristics of iDILI caused by specific drugs [41]. These statements are in line with previous recommendations [5]. To assess causality, it was outlined that RUCAM has the advantage of being objective and not requiring experts [41]. RUCAM as a diagnostic algorithm was published with the intention to improve and standardize the diagnosis of DILI by preventing the introduction of errors and subjective opinions [6,7]. This is why RUCAM is appreciated throughout the world [1,6,7,8], privileged as a structured, transparent, user friendly, objective, quantitative diagnostic algorithm [6,7,8] and specific for hepatic injury caused by drugs and herbs [8]. The updated RUCAM is intended for iDILI causality assessment of clinical trials and postmarketing evaluations by stakeholders, risk factor and mechanistic studies, case reports, epidemiology and database, registry and regulatory analyses [8], as evidenced by the 81,856 iDILI cases published recently [1] and earlier [3,8,14].

RUCAM is based on seven domains comprising key elements that are defined and provide individual scores [8]. Among the RUCAM domains, are the time to onset from the beginning (or the cessation) of the drug use (scores +2 or +1), course of ALT/ALP after cessation of the drug (scores +3 to −2), risk factors (scores +1 or 0), concomitant drug(s) (scores 0 to −3), search for alternative causes (scores +2 to −3), knowledge of product hepatotoxicity (scores +2 to 0) and response to unintentional re-exposure (scores +3 to −2) (8). The score range reflects the variability of some criteria and allows for a selection of a precise attribution, avoiding a black or white choice. With +14 down to −9 points, the final score by drugs indicates the causality level: score ≤0, excluded causality; 1–2, unlikely; 3–5, possible; 6–8, probable; ≥9, highly probable.

## 11. Overview of Individual and Combined Risk Factors

The present analysis is focused on individual and combined risk factors, not on global risks of drugs implicated in iDILI cases that can be assessed through epidemiological studies. For this approach, the total number of prescribed or sold doses are required in conjunction with the iDILI cases among the population caused by the drug and assessed for causality with the updated version of RUCAM [8]. Outside of this analysis with focus on metabolic risk factors (Figure 1 and Table 1, Table 2, Table 3, Table 4, Table 5 and Table 6) are studies on specific mechanistic steps [4,5,41], genetic risk factors [42] and results obtained from ex vivo human b liver models [43].

Risk minimizing of iDILI is a topic highlighted in the regulatory, clinical and manufacturers objectives with several but mostly unproven approaches (Table 1). In fact, there should be more research for minimizing the risk of iDILI, for instance, by using chemicals as potential drugs that do not undergo metabolism leading to ROS production. Hypotheses have been made that drugs containing a carboxylic acid functional group may be associated with a low risk of idiosyncratic drug reactions, conditions certainly requiring evaluation in a clinical iDILI setting [44] with cases assessed by the updated RUCAM [8]. The possible low risk was assumed on the basis that most drugs that contain a carboxylic acid group are metabolized to acyl glucuronides ready to undergo biliary or renal excretion [41].

Several attempts were published identifying possible individual risk factors of iDILI (Table 1). Although risk factors may tentatively be promising, confirmation by new studies with a rigorous study protocol and clear inclusion criteria of iDILI cases are needed. Many proposals were based on problematic study protocols, iDILI cases and drug or iDILI databases (Table 1). In particular, of concern are iDILI cases included in databases if they contain cases with a possible causality grading only or are not established iDILI cases like in the LiverTox database [39]. However, one of the major weaknesses is the lacking assessment for causality of the iDILI cases under consideration by a robust CAM like RUCAM in a clinical database (Table 1). RUCAM in its original version [6,7] and as an update [8] has been used in 81,856 DILI cases and 14,029 HILI (herb induced liver injury) cases all over the world [1] and outperforming in terms of case number all other CAMs [45]. RUCAM can easily be handled [46,47,48] and is commonly recommended by experts [29]. These omissions and shortcomings led to not convincing results on the identification of risk factors (Table 1).

Few reports included studies on the combination of risk factors [9,24,25]. Such results may again be considered as preliminary due to methodological issues. For instance, a high risk for iDILI was found by various combinations: first, high daily drug dose combined with drugs metabolized via CYP pathways [9]; second, high daily drug dose combined with high hepatic drug metabolism [22], but this combination was considered not to be more predictive of iDILI than using daily drug dose or liver metabolism alone [25]; third, daily drug dose combined with lipophilicity [24], but this combination appeared ineffective in a larger number of drugs [9,25] although appreciated in a commentary highlighting that both are better than one to avoid iDILI [49].

In addition to the comments presented above and included in Table 1, studies on the association between iDILI and daily drug dose, liver metabolism and drug lipophilicity were critically analyzed [25]. In more detail, it was outlined that the general belief remains that none of these medication characteristics are able to predict iDILI with high confidence, leading to the statement of an outstanding caveat. It was outlined that all previous studies lacked comprehensiveness, that is, the number of drugs examined is relatively small as compared with all FDA approved new molecular entities or currently used oral prescription drugs [25]. It seems that the present controversy around risk factors is fairly limited to scientists of the FDA [9,24,25,26], an interesting constellation calling for solutions in domo.

## 12. Conclusions

The most critical feature of iDILI is its unpredictability. This clinical issue stimulated experts to search for risk factors to minimize this risk. Proposed risk factors like recommended daily drug doses, actually used daily drug doses, hepatic drug metabolism and drug lipophilicity are certainly insufficient due to major methodological problems leading to serious caveats. Weaknesses include low case numbers, unclear inclusion criteria of cases, lack of correct definition of the liver injury and iDILI cases not evaluated by a robust causality assessment method such as RUCAM, whatever the data source is. However, an important feature is for the first time, on a quantitative basis, most drugs (61.1%) implicated in causing 3312 iDILI assessed with RUCAM, were found metabolized in pathways involving cytochrome P450 (CYP) isoforms: 49.6% were metabolized by CYP 3A4/5, 24.6% by CYP 2C9, 13.2% by CYP 2E1, 7.3% by CYP 2C19, 3.5% by CYP 1A2 and 1.8% by CYP 2D6. In conclusion, despite major methodological shortcomings involving various parameters, the conclusion was reached that risk factors were found, at least tentatively, for drugs inducing iDILI metabolized by specific CYP isoforms. More studies in this area are needed avoiding the described weaknesses and following rigorous protocols including methods to identify properly real cases of iDILI.

## Figures and Tables

**Table 1:** Listing of published proposals for potential risk factors of iDILI with focus on cytochrome P450, drug dose, metabolic drug parameters and drug lipophilicity.

Proposed Risk Factors	Comments	Year	First, Author
Cytochrome P450 (CYP) CYP isoforms	**Proposal:** Described as classic view, drugs implicated in iDILI are mainly metabolized by CYP. **Comment:** In retrospect, correct assumption but not based on facts or iDILI cases assessed for causality like by RUCAM although this method was quoted. **Proposal:** Officially proposed, most hepatotoxic drugs are oxidatively metabolized by the CYP system. **Comment:** This sentence statement was not based on any iDILI case assessed for causality by a robust diagnostic algorithm like RUCAM. **Proposal:** In general, CYP 2C9 and CYP 2 C19 pathways appeared more toxic than CYP 3A and CYP 2D6. Data were retrieved from various databases. **Comment:** Conclusions remain vague because iDILI cases were likely not assessed for causality by RUCAM. **Proposal:** Being a substrate of CYP enzymes is one of two important predictors of iDILI, having a higher, dose-independent likelihood of causing iDILI based on the adjusted OR (odds ratio) of 5.04 with 95% confidence interval (CI) of 2.34–10.9, *p* <0.0001. Concomitantly, drugs functioning as CYP inhibitor or inducer had no statistically significant effect on OR. **Comment:** Metabolism data on CYP enzymes for 254 orally administered drugs were collected from the US Liver Toxicity Knowledge Base Benchmark Dataset, but quality details of the used iDILI cases were not provided, lacking especially information whether RUCAM was used for causality assessment or not. Therefore, these data may be considered as preliminary, applicable also to data on CYP isoforms: drugs metabolized by CYP 1A2, CYP 2C8/2C9 and CYP 3A5 are classified having a higher likelihood of causing iDILI as compared with CYP 2D6, CYP 2C19, CYP 3A4, CYP 2B6, and CYP 2E1. Presented were also data of 11 clinical iDILI cases derived from the LiverTox database with causality assessment confirmed by physicians or health care professionals. Many of the assumed iDILI cases of this database were not real iDILI and became a matter of dispute. Other iDILI were derived from Liver Toxicity Biomarker Study, lacking details on causality assessment and biomarkers used. **Proposal:** As opposed to the proposals made above, recent reports provided clarity on the relationship between drugs implicated in iDILI: Among 36 drugs causing iDILI in published cases assessed for causality using RUCAM, 22 drugs (61.1%) were metabolized through CYP, whereas for the remaining 14 drugs (38.9%) pathways not involving CYPs were responsible for the drug metabolism. As a result, CYP is involved in the majority of drugs implicated in iDILI. Among 619 RUCAM based published iDILI cases caused by drugs metabolized by CYPs, 49.6% of the cases were due to drugs metabolized by CYP 3A4/5, with 24.6% cases by CYP 2C9, 13.2% cases by CYP 2E1, 7.3% cases by CYP 2C19, 3,5% cases by CYP 1A2, and 1.8% cases by CYP 2D6. **Comment:** For the first time, the percentage contribution of CYP and CYP isoform was determined in iDILI cases assessed for causality by RUCAM.	2009 2009 2010 2014 2020	Tarantino [18] US FD A see [9] Lammert [22] Yu [9] Teschke [21]
Recommended daily drug dose (RDDD)	**Proposal:** A relationship was assumed between recommended, not necessarily used, daily drug doses of oral medications with iDILI. The US study cohort retrieved the cases and the used drugs including their recommended daily doses from two publicly available pharmaceutical databases. The drugs were categorized into dosage groups of 10 mg or less, 11 to 49 mg and 50 mg or greater based on daily recommended doses, which originally may show a broad range not allowing for inclusion in a precise dosage group. With atorvastatin as an example for a problematic dosage group attribution, the recommended daily dose was described as ranging between 10 and 80 mg. Among US prescription medicines, a statistically significant relationship was observed between recommended daily drug dose and reported frequency of hepatic adverse events like liver failure, liver transplantation and death caused by iDILI. **Comment:** The proposed daily drug dose dependency of iDILI was not based on the actually used daily dosage but on drugs with a broad dosage range as recommended by the manufacturers. Problematic was also the selection of iDILI cases likely not assessed for causality using RUCAM. Therefore, problems of dosages and iDILI cases limit the validity of published conclusions, classifying the data at best as preliminary. **Proposal:** High recommended daily drug doses of ≥100 mg daily were assumed as risk factors of iDILI, based on an OR of 6.92 and a 95% CI (3.10–15.63). Data were retrieved from several sources of likely variable, not further described quality. For instance, iDILI cases were used included in the LiverTox database, which is known for presenting many iDILI cases without confirmed causality, causing iDILI cases being non-iDILI cases. Apart from high doses, lipophilicity of the drugs was considered to contribute to the risk of liver injury. **Comment:** The vague quality of the used iDILI cases substantially limits the published conclusions. **Proposal:** A high daily drug dose, as recommended by the manufacturers but not necessarily as used dose, was considered as one of two important predictors of iDILI. **Comment:** Data were taken from the US Liver Toxicity Knowledge Base Benchmark Dataset, containing iDILI cases likely not assessed for causality using RUCAM. Recommended doses were not necessarily doses actually used. Overall data of insufficient quality caused fragile conclusions. **Proposal:** A higher recommended daily drug dose was considered as risk factor for iDILI. Basic data were derived from the World Health Organization’s Anatomical Therapeutic Chemical classification system and the Micromedex Drugdex® compendium. **Comment:** The quality of the data retrieved from other sources remained unclear, Data were based on recommended but not on actually used drugs and iDILI cases were obviously not assessed for causality using RUCAM. These confounders limit the conclusions as published.	2008 2013 2014 2015	Lammert [23] Chen [24] Yu [9] Weng [25]
Used daily drug dose (UDDD)	Proposals: Published in 1999, drugs given at a daily dose of 10 mg or less were rarely or if ever associated with a high incidence of idiosyncratic drug reactions (iDRs), mentioned in the context of published data on the lupus syndrome induced by hydralazine and the increase in mortality with vesnarinone, both conditions that have nothing to do with iDILI. Published 2007, it was claimed as an empirical observation that that iDRs are rare with drugs given at a dose of 10 mg daily or less, whereby iDILI is not mentioned. In 2019, if a drug is given at a total daily dose of 10 mg/day or less, it is unlikely to be associated with a significant incidence of iDILI, considered as an empirical cutoff. The problem with this cutoff remains because it is not based on an iDILI case study. **Comment:** Only in 2019, a drug threshold was attributed to iDILI but without providing new evidence. **Proposal:** Swedish cohort: included were 598 iDILI cases, 9% belonged to the ≤10 mg/day group, 14.3% to the 11–49 mg/day group and 77% of cases were caused by medications given at doses ≥50 mg/day. Cases were retrieved from the Swedish Drug Reaction Advisory Committee (SADRAC) and assessed for causality using RUCAM and all included cases had a causality grading of at least possible. **Comment:** The percentage contribution of cases with a possible causality grading was not mentioned, in other studies using iDILI cases of SADAC up to 48% of the cases had a possible causality grading. Therefore, the present data should be taken with caution, because it remains unclear how many cases had the preferred causality gradings of highly probable or probable. It is also unclear how many real liver injury cases were included with alanine aminotransferase (ALT) values of >5 x ULN (upper limit of normal) and whether cases with ALT values of 2 – 5 x ULN were excluded from the study cohort as not real DILI cases.	1999 2007 2019 2008	Uetrecht [20] Uetrecht [19] Uetrecht [4] Lammert [23]
Cumulative drug dose (CDD)	**Proposal:** There are no valid data proposing that cumulative drug doses could be risk factors of iDILI. **Comment:** This is a major shortcoming in previous risk factor studies on iDILI. However, for a few drugs implicated in iDILI sufficient data were presented for used daily doses and duration of intake, allowing now for calculation of the cumulative drug doses, see below. They ranged from 575 mg to 12,000 mg and provided no trend and no realistic threshold that could be used in the context of risk management In addition, case data were retrieved from the Swedish Hepatic ADR Dataset, known for its problems of correct iDILI definition. **Proposal:** The potential role of cumulative drug doses for iDILI remained unanswered. **Comment:** However, based on published data of used daily drug doses in 6 patients with length of treatment, cumulative doses were now calculated as shown below and provided cumulative doses from 3200 mg to 60,000 mg. Interestingly, the 60,000 mg was used by patient 6, who recovered. Overall, a valid trend was not found.	2008 2013	Lammert [23] Chen [24]
Hepatic drug metabolism (HDM)	**Proposal:** Oral medications with significant hepatic metabolism are at higher risks for hepatic adverse events. Compared to drugs with <50% hepatic metabolism, drugs with ≥50% hepatic metabolism had a significantly higher frequency of liver failure (28% vs. 9%), fatal DILI (23% vs. 4%). **Comment:** Cases of iDILI and drug characteristics were retrieved from a number of databases with variable quality, and there were no strict inclusion criteria of cases and no causality assessment such as RUCAM. Cases with ALT values above 3 x ULN rather than above 5 x ULN were used, a problematic approach due to inclusion of iDILI cases that were not real liver injury cases. **Proposal:** Liver metabolism ≥50% was found to be predictive of fatal iDILI lacking synergistic effects with any other risk factor. The study was based on data of the WHO Anatomical Therapeutic Chemical classification system and the Micromedex Drugdex® compendium. **Comment:** The main problem was the obvious lack of a rigorous causality assessment method applied to iDILI cases, which makes this methodological shortcoming the published claims not well evidenced. **Proposal:** A hepatic metabolism ≥50% provided for ORs (95%CI): 1.90 (1.18–3.5), classified under the term “consensus” as risk factor for iDILI, although it was admitted that the extent of metabolism is not a strong predictor for iDILI risk. Data were retrieved from various databases including the US LiverTox database despite its known limitations. **Comment:** It was correctly mentioned by the authors as one of several limitations that not all of the datasets considered the causality assessment (e.g., RUCAM score), which they found essential in future studies to characterize iDILI, as reports suggested that some hepatotoxicity recorded in the literature is vague. The authors described other limitations with respect to the used databases.	2010 2015 2017	Lammert [22] Weng [25] McEuen [26]
Drug lipophilicity (DL)	**Proposal:** High lipophilicity per se is not a significant risk factor for iDILI, opposing to the title of the publication: High lipophilicity and high daily dose of oral medications are associated with significant risk for drug-induced liver injury. **Comment:** In fact, lipophilicity alone is not a significant risk factor for iDILI, only if combined with high daily doses. For lipophilicity an OR of 1.83 was calculated, a value that was not statistically significant. **Proposal:** High drug lipophilicity is not associated with a higher likelihood of causing iDILI. **Comment:** It was correctly mentioned by the authors that data for drug lipophilicity were derived from 254 orally administered drugs collected from the Liver Toxicity Knowledge Base Benchmark Dataset. However, quality details of the used iDILI cases were not provided, lacking especially information whether RUCAM was used for causality assessment or not. Therefore, these data may be considered as provisional. **Proposal:** According to the published conclusions, it seems that lipophilicity does not play a significant role in predicting a liver risk by an oral drug. **Comment:** These negative results were based on databases and 975 oral medications. **Proposal:** For lipophilicity as a potential risk factor for iDILI, a consensus among the authors was reached regarding the low OR (95% CI) of 1.55 (109–2.22). For analysis, databases were used including the problematic US LiverTox database, and not all of the iDILI cases were assessed for causality using RUCAM. **Comment:** Results concerning lipophilicity as risk factor of iDILI remain fragile.	2013 2014 2015 2017	Chen [24] Yu [9] Weng [25] McEuen [26]

**Table 2:** Ranking of drugs causing iDILI with causality assessment of cases by RUCAM. Modified from previous publications [3,5,21]. Listed are the top ranking 48 drugs implicated in causing 3312 iDILI cases with verified causality using RUCAM [3]. The predominant CYP isoforms but not minor isoforms involved in drug metabolism are listed, with references provided in an earlier report [5,21]. CYP isoforms and nonCYP pathways were derived from clinical and experimental studies, as mentioned in original reports and published listings. Abbreviations: CYP, Cytochrome P450; DILI, Drug induced liver injury: NA, not available.

Drug	*RUCAM Based DILI Cases (n)*	*Metabolized by CYP Isoform*
1. Amoxicillin-clavulanate	333	-
2. Flucloxacilllin	130	CYP 3A4
3. Atorvastatin	50	CYP 3A4/5
4. Disulfiram	48	CYP 2E1
5. Diclofenac	46	CYP 2C8
6. Simvastatin	41	CYP 3A4/5
7. Carbamazepine	38	CYP 3A4/5
8. Ibuprofen	37	CYP 2C8/9
9. Erythromycin	27	CYP 3A4
10. Anabolic steroids	26	CYP 2C19
11. Phenytoin	22	CYP 2C9
12. Sulfamethoxazole/Trimethoprim	21	CYP 2C9
13. Isoniazid	19	CYP 2E1
14. Ticlopidine	19	CYP 2C19
15. Azathioprine/6-Mercaptopurine	17	-
16. Contraceptives	17	CYP 3A4
17. Flutamide	17	CYP 1A2
18. Halothane	15	CYP 2E1
19. Nimesulide	13	CYP 2C9
20. Valproate	13	CYP 2C9
21. Chlorpromazine	11	CYP 2D6
22. Nitrofurantoin	11	-
23. Methotrexate	8	-
24. Rifampicin	7	-
25. Sulfazalazine	7	-
26. Pyrazinamide	6	-
27. Natriumaurothiolate	5	-
28. Sulindac	5	CYP 1A2
29. Amiodarone	4	CYP 3A4
30. Interferon beta	3	-
31. Propylthiouracil	2	CYP/NA
32. Allopurinol	1	-
33. Hydralazine	1	-
34. Infliximab	1	-
35. Interferon alpha/ Peginterferon	1	-
36. Ketaconazole	1	-
37. Busulfan	0	-
38. Dantrolene	0	-
39. Didanosine	0	-
40. Efavirenz	0	CYP 2B6
41. Floxuridine	0	-
42. Methyldopa	0	CYP/NA
43. Minocycline	0	-
44. Telithromycin	0	CYP 3A4
45. Nevirapine	0	CYP 3A4
46. Quinidine	0	CYP 3A4
47. Sulfonamides	0	CYP/NA
48. Thioguanine	0	-

**Table 3:** Ranking of CYP isoforms involved in drug metabolism of patients with RUCAM based iDILI [3,5,21] as compared with the general population without iDILI [12]. Data were partially modified from previous publications [3,5,21]. Listed are drugs implicated in 619 RUCAM based iDILI cases and metabolized by CYP isoforms, data are given in number for drugs(n) and in percentages (%) as metabolized by CYP isoforms. These data were compared with the contribution of CYP isoforms in the general population without iDILI [12]. Abbreviations: CYP, Cytochrome P450; iDILI, idiosyncratic drug induced liver injury.

CYP Isoform.	Drugs Implicated in RUCAM Based iDILI Cases and Metabolized by CYP Isoforms (n)	Drugs Implicated in RUCAM Based iDILI Cases and Metabolized by CYP Isoforms (%)	Contribution of CYP Isoforms in Drug Metabolism in the General Population (%)
CYP 1A2	22	3.5	5
CYP 3A4/5	307	49.6	40–45
CYP 2C9	152	24.6	10
CYP2 C19	45	7.3	5
CYP 2D6	11	1.8	20–30
CYP 2E1	82	13.2	2–4

**Table 4:** Listing of CYP isoforms as risk factors for drugs implicated in causing iDILI. Data are derived from published reports [9,21,22]. Some cases of iDILI were assessed for causality using RUCAM [21,22], others were not assessed [9]. Abbreviations: CYP, Cytochrome P450; iDILI, idiosyncratic drug induced liver injury; NA, not available; RUCAM, Roussel Uclaf Causality Assessment Method.

Risk Level	CYP Isoform as Risk Factor for Drugs Implicated in Causing iDILI		
High risk ▼ Low risk	**Report 1 [21]**	**Report 2 [9]**	**Report 3 [22]**
	CYP 3A4/5	CYP 1A2	CYP 2C9
	CYP 2C9	CYP 3A5	CYP 2C19
	CYP 2E1	CYP 2C8	CYP NA
	CYP 2C19	CYP 2C9	CYP NA
	CYP 1A2	CYP 2C19	CYP 2D6
	CYP 2D6	CYP 3A	
		CYP 2B6	
		CYP 2E1	

**Table 5:** Role of cumulative doses of drugs implicated in causing iDILI. Results were calculated from data presented in an earlier study [23]. Data were derived from patients, who used the drugs at a dose of <50 mg daily as documented in the Swedish Hepatic ADR Dataset and experienced a poor outcome of iDILI like death or liver transplantation. Abbreviation: ADR, Adverse Drug Reaction.

Patient	Drug	Daily Dose	Duration of Exposure (Days)	Cumulative
		(mg/Day)		Dose (mg)
1	Donepezil	5	501	2505
2	Enalapril	10	60	600
3	Omeprazole	40	300	12,000
4	Simvastatin	20	90	1800
5	Rofecoxib	25	23	575
6	Enalapril	30	60	1800
7	Simvastatin	20	90	1800
8	Hydralazine	25	180	4500
9	Dikumarol	40	120	4800

**Table 6:** Role of cumulative doses of drugs implicated in causing iDILI. Results were calculated from data presented in an earlier study (37). Clinical presentation was acute liver failure in patients 1–3, acute hepatitis in patient 4, acute hepatitis and jaundice in patient 5 and acute cholestatic hepatitis in patient 6. All patients were treated with a multidrug regime, consisting of up to 6 additional drugs. Abbreviation: LTX, Liver transplantation.

Patient	Drug	Daily Dose	Duration of Exposure (Days)	Cumulative	Outcome
		(mg/Day)		Dose (mg)	
1	Tolcapone	200	60	12,000	Death
2	Diclofenac	150	42	6300	Death
3	Ketoconazole	200	58	11,600	LTX
4	Fenofibrate	300	14	4200	Recovery
5	Disulfiram	100	32	3200	Recovery
6	Ticlopidine	500	120	60,000	Recovery

## Data Availability

Data were derived from published reports.
